# SARS-CoV2 and Co-Infections: A Review of Two Cases

**DOI:** 10.1155/2020/8882348

**Published:** 2020-09-14

**Authors:** Sumit Sohal, Guillermo Rodriguez-Nava, Ramzy Khabbaz, Sana Chaudry, Clio Musurakis, Solab Chitrakar, Vishnu V. Chundi, Harvey J. Friedman

**Affiliations:** ^1^Department of Internal Medicine, AMITA Health Saint Francis Hospital, 355 Ridge Avenue, Evanston, IL 60202, USA; ^2^Department of Infectious Diseases, AMITA Health Saint Francis Hospital, 355 Ridge Avenue, Evanston, IL 60202, USA; ^3^Department of Pulmonary Medicine and Critical Care, AMITA Health Saint Francis Hospital, 355 Ridge Avenue, Evanston, IL 60202, USA

## Abstract

COVID-19 infection caused by SARS-CoV2 virus is an acute respiratory illness which was declared as a pandemic by the World Health Organization. Usually, SARS-CoV2 infects independently and can cause spectrum of disease ranging from mild illness to severe progressive pneumonia, multiorgan dysfunction, and death; however, co-infections with other respiratory pathogens have been noted. Here, we present 2 fatal cases with co-infection, one with parainfluenza-4 virus and other co-infection/secondary infection with *Streptococcus pneumoniae* bacteria. Further studies are needed to study the effect of co-infections on morbidity and mortality of patients and establish the outcome of such infections.

## 1. Introduction

COVID-19 is an acute respiratory illness caused by SARS-CoV2 virus which was first recognized in December 2019 in Wuhan, China [1]. On March 11, 2020, the World Health Organization declared the COVID-19 outbreak a pandemic [2]. The full spectrum of Covid-19 ranges from mild, self-limiting respiratory tract illness to severe progressive pneumonia, multiorgan failure, and death [3]. Patients with COVID-19 may also have co-infection with other seasonal respiratory pathogen, and similar dual respiratory viral illnesses have been studied in the past, reporting higher rates of hospitalizations than with patients with single viral illness [4, 5]. Co-infection with several viruses has been documented, especially with influenza A or influenza B, but here we present 2 rare co-infection cases, one with parainfluenza-4 virus and the other with *Streptococcus pneumoniae*.

## 2. Case Description

### 2.1. Case 1

**Figure 1:**
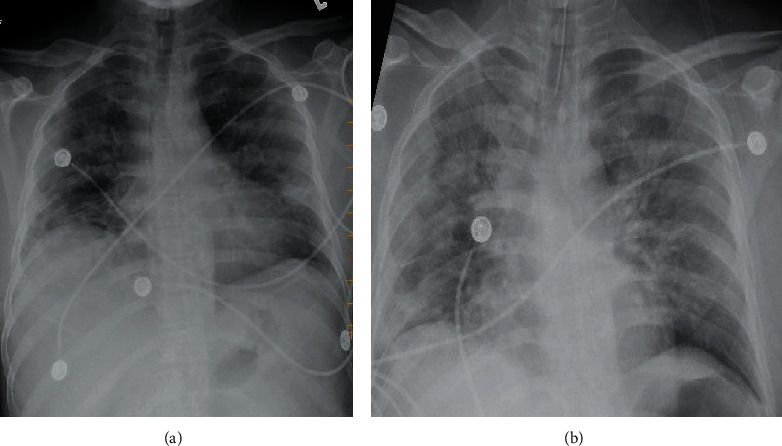
(a) Multifocal interstitial opacities throughout the lung parenchyma bilaterally with elevated hemidiaphragm, discoid atelectasis, and volume loss noted in the right lower lobe. (b) Diffuse interstitial opacities that have increased as compared with the previous exam with endotracheal tube approximately 4 cm from carina.

This case is of a young male in his 40s with no significant medical history who presented with fever. Patient reported fever 1 week prior to admission which was accompanied by cough and generalized body aches. He described his cough as mostly dry with occa-sional phlegm without blood. He also described development of headache 3 days prior to presentation which was followed by shortness of breath 2 days prior to admission and came to Emergency Room (ER) when it got worsened. He denied any gastrointestinal complains or any sick contacts. Upon arrival to ED, he was noted to have a fever of 101 F, with HR of 99 bpm, RR of 28 breaths per min, and saturating 99% on 4 lpm nasal cannula. The patient was admitted to the ICU, and the nasopharyngeal swab to rule out COVID-19 infection along with other respiratory viral pathogens was sent. Several laboratory parameters including some inflammatory markers were also sent (Table 1). A chest X-ray was done, and it is shown in Figure 1(a). He was started on ceftriaxone and azithromycin.

The patient deteriorated with increased work of breathing, tachypnea, and use of accessory muscles of breathing, following which the patient was intubated. During intubation, patient's airways were described as erythematous and edematous especially with angry-looking epiglottis. He was placed on assist/control mode of mechanical ventilation at a rate of 24, with a tidal volume of 400, FiO_2_ of 100%, and PEEP of 10, and a chest X-ray was obtained again after intubation (Figure 1(b)). PCR for respiratory viral pathogens tested positive for parainfluenza-4 virus, and on the subsequent day, he was also reported to have a positive PCR for SARS-CoV2. The patient remained intubated for several days before going into cardiac arrest and expired after 9 days of hospitalization.

### 2.2. Case 2

**Figure 2:**
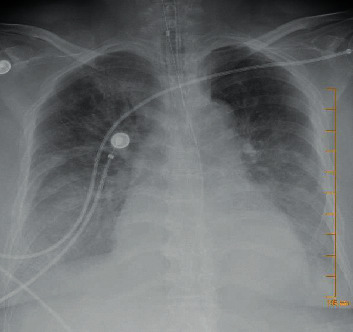
Bilateral interstitial and intra-alveolar infiltrates with small bilateral pleural fluid.

The patient is an elderly female in her 80s with a medical history of diabetes mellitus, hypertension, asthma, paroxysmal atrial fibrillation and dementia who was brought to the hospital after a cardiac arrest. Initial rhythm was reported to be pulseless electrical activity (PEA) and return of spontaneous circulation was achieved after 30–40 minutes. The patient was immediately intubated on arrival and reported a temperature of 98.4 F, HR of 57, BP of 85/47 mm of Hg, and RR of 14, being ventilated with assist/control mode set at 14 breaths per min, with a tidal volume of 400 and FiO_2_ of 60% at a PEEP of 5. The patient was started on vasopressors, norepinephrine. Laboratory values are outlined in Table 1, and X-ray is shown in Figure 2. The respiratory viral panel, nasopharyngeal swab for SARS-CoV2, and urine antigens were sent. The patient was resulted positive for SARS-Cov2, and later in the day, urine antigen also came back positive for *Streptococcus pneumoniae*. The patient was treated with ceftriaxone, azithromycin, and hydroxychloroquine. After several days of intubation with no signs of neurological recovery, the family requested for withdrawal of care and she eventually passed away.

## 3. Discussion

As of April 30, 2020, more than 3,250,000 cases of COVID-19 have been documented with more than 233,250 deaths worldwide, with USA alone contributing to more than 1,000,000 cases with over 63,000 deaths since the start of the pandemic [6]. Most of the data of co-infection comes from China where several case reports and case series have been published. Xing et al. described the difference in rate of co-infection in patients with COVID-19 between the cities of Qingdao and Wuhan with Qingdao reporting 80% patients with co-infection whereas Wuhan with only 2.63% patients with co-infection. Influenza A was the highest co-infectant [5]. Another article by Ding et al. described the clinical characteristics of patients with COVID-19 and influenza co-infection [7]. Wang et al. reported that 5.8% of 2019-nCoV-infected patients had other pathogen infections [8] Xing et al. did not report significant different clinical manifestations or disease prognosis in the two disease groups in these two different cities, and similarly, none of the patients in study by Ding et al. required ICU stay [5, 7]. Xing et al. reported no case of parainfluenza virus co-infection [5]. A case series from Iran also reported a COVID-19 and influenza co-infection. 2 other independent case reports have been reported on human meta-pneumovirus and mycoplasma pneumoniae co-infection, respectively [9, 10]. Cases of COVID-19 and parainfluenza-4 virus co-infection are very rare. Cases of co-infection have been mentioned in a report by Stanford Medicine Data Scientists [11] and in the study by Wang et al. [8].

Bacterial infections (co-infections and secondary bacterial infections) are common in patients with viral pneumonia and have been seen in patients with COVID-19 pneumonia. The histological and pathological evidence during influenza pandemic of 1918-19 suggests that majority of deaths occurred from secondary bacterial infections. Several studies in 1940s on animals suggested that influenza virus acted synergistically with several pneumopathic bacteria to produce either a higher incidence of disease or death rate [12, 13]. Similar observations were also seen in H1N1 influenza infection of 2009 with *Streptococcus pneumoniae* being the most common bacteria [12, 14]. Experimental studies in the past have shown that inflammation caused by influenza can cause decreased innate immune control of pneumococcus and thus leading to worse outcomes [15, 16]. Cell breakdown caused by viral pathogens damages the mucocilliary barrier leading to bacterial spread [13]. Thus, use of empirical antibiotics is common in these patients, and various studies recommend the use of antipneumococcal and antistaphylococcal antibiotics especially in patients with deteriorating clinical status [8, 16].

Several outcomes of viral co-infections have been noted in the literature; however, co-infections of certain viruses may promote an increase in viral replication [17]. An interesting phenomenon was studied by Goto et al. where human parainfluenza virus 2 infection-associated cell fusion facilitated influenza A virus replication and modulated pathological consequences [17, 18]. Similarly, viral co-infections can also alter the disease severity, and in the experimental model, SAR-CoV and reovirus co-infections have shown worsened outcomes in guinea pigs [17, 19].

Though studies for COVID-19 till date have not shown any difference in outcomes in patients with co-infection, year-old studies have shown increased rate of hospitalizations in patients with dual viral illnesses [4, 5]. Co-infection with bacterial pathogens can increase the likelihood of severe illness.

## 4. Conclusion

Co-infections can be noted in COVID-19 and may impact outcomes of the patients; however, the degree of impact is unknown. Historical data from other viruses suggest worse outcomes; however, data on COVID-19 are still accumulating, and case reports and series are being reported frequently. Further review of these co-infections is needed to study the epidemiology and clinical and laboratory characteristics of this subgroup of patients and assess the effect of co-infection on outcomes of these patients.

## Figures and Tables

**Table 1:** Comparison of laboratory variables of case 1 and case 2.

Variables	Case 1	Case 2	Reference
White blood count	9.9	34.7	4.0–11.0 k/mm cu
Serum procalcitonin	0.75	4.41	0.20–0.49 ng/ml
Serum LDH	392	2335	140–271 IU/L
Serum ferritin	1603	1301	24–336 ng/ml
C-reactive protein	28.2	0.4	0.0–0.9 mg/dl
D-dimer	457	5755	0–622 ng/ml

## Data Availability

The laboratory and radiological variables and outcome data for both patients will be available upon request.
